# Effect of dexmedetomidine on prevention of postoperative nausea and vomiting in pediatric strabismus surgery: a randomized controlled study

**DOI:** 10.1186/s12886-020-01359-3

**Published:** 2020-03-05

**Authors:** Shuangshuang Li, Tingjie Liu, Junming Xia, Jie Jia, Wenxian Li

**Affiliations:** Department of Anesthesiology, Eye & ENT Hospital of Fudan University, 83 Fenyang Road, Shanghai, 200031 China

**Keywords:** Dexmedetomidine, Postoperative nausea and vomiting, Strabismus surgery, Oculocardiac reflex, Emergence agitation

## Abstract

**Background:**

Postoperative nausea and vomiting (PONV) are common side-effects following strabismus surgery. The present study aimed to compare the effects of different doses of dexmedetomidine (DEX) on PONV incidence in pediatric patients undergoing strabismus surgery.

**Methods:**

In this prospective randomized double-blinded study, 126 pediatric patients undergoing strabismus surgery were randomized into one of three groups: Placebo group, normal saline; DEX1 group, 0.3 μg/kg dexmedetomidine, and DEX2 group, 0.5 μg/kg dexmedetomidine. Oculocardiac reflex (OCR) events were recorded during surgery. PONV or postoperative vomiting (POV) was recorded for 24 h in the ward. Pediatric anesthesia emergence delirium (PAED) scale and emergence agitation (EA) scale were recorded in the recovery room.

**Results:**

Intraoperative OCR was significantly reduced in DEX2 group (42%) as compared to that of Placebo group (68%) (*p* = 0.0146). During the first 24 h post-op, the overall incidence of PONV was significantly lower in DEX2 group (10%) than that of Placebo group (32%) (*p* = 0.0142). There was no significant difference in POV among the three groups. PAED or EA scores among the three groups were similar during recovery time.

**Conclusion:**

Dexmedetomidine (0.5 μg/kg) reduced OCR and PONV without lengthening extubation time or recovery time in pediatric patients undergoing strabismus surgery.

**Trial registration:**

The trial was prospectively registered before patient enrollment at Chinese Clinical Trial Registry (Clinical Trial Number: ChiCTR1800020176, Date: 12/19/2018).

## Background

Although often considered a minor side effect, postoperative nausea and vomiting (PONV) are common unpleasant adverse effects after surgery and may increase patient discomfort, result in serious complications, and delay patient discharge. The incidence of PONV in pediatric patients is often twice as high as in adults [1]. There are four main risk factors for PONV or POV in pediatric anesthesia: duration of surgery longer than 30 min, age ≥ 3 years, previous PONV or a positive family history, and strabismus surgery [2]. Strabismus surgery is an independent risk factor for PONV in pediatric patients. Previous studies have reported that the incidence of PONV is as high as 37–80% after strabismus surgery under general anesthesia [3]. Routine efforts to prevent PONV include butyrophenones, benzamides, histamine and muscarinic receptor antagonists, corticosteroids, and serotonin 5-HT3 receptor antagonists [4–7]. Despite numerous efforts to decrease the incidence of PONV, it remains a significant challenge in pediatric patients undergoing strabismus surgery.

Dexmedetomidine is a highly selective α2-adrenoreceptor agonist which has been widely used in clinical practice [8] and has been explored extensively in the pediatric population due to its beneficial effects on perioperative morbidities [9]. In the past few years, many studies in pediatrics have been published showing that dexmedetomidine lowered postoperative pain scores and opioid consumption, decreased the incidence of emergence agitation (EA) and improved the quality of recovery in pediatric patients undergoing different surgical procedures [10–12]. In addition, a small selection of studies reported that dexmedetomidine could lower the incidence of nausea or vomiting after surgery and during the use of patient-controlled analgesia (PCA) in pediatrics [13, 14]. However, the effect of dexmedetomidine on PONV remains poorly understood.

In order to address these knowledge gaps, the present study was conducted to investigate the effects of two different doses of dexmedetomidine on the incidence of PONV in pediatric patients undergoing strabismus surgery.

## Methods

### Study population

Ethical approval was obtained from the Ethics Review Committee of the Eye, Ear, Nose & Throat (EENT) Hospital of Fudan University along with written informed consent from the parents or legal guardians of all participants. In total, we enrolled 126 children (6 to 10 years of age, American Society of Anesthesiologists (ASA) physical status I to II) undergoing strabismus surgeries between December 2018 and March 2019 at the EENT Hospital, Shanghai, China. Primary exclusion criteria included children with known gastroesophageal reflux disease, intake of antiemetic or psychoactive medication within 24 h, developmental delays, and allergy to the study medications. Patients were randomized using a computer randomization program into one of three groups: Group Placebo which received normal saline, Group DEX1 which received dexmedetomidine 0.3 μg/kg (Jiangsu Xinchen Pharmaceutical Co., Ltd., Lianyungang, China), and Group DEX2 which received dexmedetomidine 0.5 μg/kg.

### Study protocol

Prior to surgery, pediatric patients fasted for more than 8 h. Pulse oximetry, electrocardiography, and non-invasive blood pressure were recorded in the operating room. After confirmation of patients’ baseline vital signs, anesthesia was induced with propofol 2 mg/kg, cis-atracurium 0.15 mg/kg and fentanyl 2.5 μg/kg. Literatures reported that 0.3–1.0 μg/kg dexmedetomidine was a commonly used dose after induction in pediatric surgeries [9]. There were several literatures studied effects of 0.3 μg/kg dexmedetomidine on emergence agitation, recovery profiles, or parents’ satisfaction in pediatric surgeries [15, 16]. In addition, previous studies reported that 1.0 μg/kg dexmedetomidine delayed eye opening, extubation time and post anesthesia care unit (PACU) stay time [17, 18]. In our pilot study, the children with 1.0 μg/kg dexmedetomidine had longer drowsiness after operation, which made the parents more anxious. Based on the above-mentioned factors, the doses of 0.3 μg/kg and 0.5 μg/kg were chosen in our study. Patients in the Group Placebo, Group DEX1 and Group DEX2 were administered normal saline, dexmedetomidine 0.3 μg/kg, and dexmedetomidine 0.5 μg/kg, respectively. The drug was administered intravenously every 10 min for each group. A laryngeal airway mask was inserted 3 mins after induction of anesthesia. IV dexamethasone 0.1 μg/kg was administered at the beginning of surgery. General anesthesia was maintained using 0.9–1.1 minimum alveolar concentration sevoflurane and 50% air in oxygen with a constant fresh gas flow of 2 L/min.

The oculocardiac reflex (OCR) was defined as an acute reduction of ≥20% in HR associated with traction on the eye muscle. The OCR event numbers were recorded during this procedure. If HR did not return to baseline after releasing the muscle, atropine (0.01 mg/kg) was injected.

Every patient received intravenously administrated droperidol before completion of the surgery. Sevoflurane administration was terminated when patients were delivered to PACU. All patients received topical anesthesia administered as two conjunctival drops of 0.4% oxybuprocaine (Laboratoire Chauvin, Aubenas, France) after closure of the conjunctiva. The flexible LMA was removed once the respiration parameters reached TV ≥ 8 mL/kg, RR ≥ 15 breaths/min, and PET_CO2_ levels of 40–45 mmHg. The incidence of bucking on removal of the LMA was recorded. The anesthesiologist performing the extubation was blinded to the treatment condition.

A well-trained PACU nurse blinded to the study groups assessed the state of consciousness using the pediatric anesthesia emergence delirium (PAED) scale and EA scale at four time points: T_0_: removing the LMA; T_1_: 5 min after removing the LMA; T_2_: 10 min after removing the LMA; T_3_: 15 min after removing the LMA [19, 20]. The PEAD scale contains five items: eye contact, purposefulness of actions, awareness of surroundings, restlessness and consolability. Each item was scored into five grades (0–4) according to degree, giving a maximum total of 20 points. The EA scale contains five grades (1 = sleeping, 2 = awake and calm, 3 = irritable and crying, 4 = inconsolable and crying, and 5 = severe restlessness and thrashing). Patients with severe agitation defined as a PAED score ≥ 10 or EA score ≥ 4 were treated with intravenous propofol (1 mg/kg).

All episodes of nausea or vomiting were recorded by a well-trained PACU nurse during the 24 h following return to the ward. Pediatrics who experienced more than two emetic episodes and those who requested an antiemetic were treated with ondansetron 0.1 mg/kg. A well-trained PACU nurse asked about the degree of pain and recorded answers using a visual rating scale (VRS) with options from 0 (not painful at all) to 10 (extremely painful) at four time points: T_00_: out of PACU; T_11_: 4 h after operation; T_22_: 12 h after operation; T_33_: 24 h after operation. If patients complained of severe pain (VRS > 4), 0.5 μg/kg fentanyl was administered.

### Statistical analysis

The incidence of PONV during the 24-h period after surgery was the primary outcome measured in this study. Based on a published PONV incidence of 70% in strabismus surgery, a sample size of 38 patients per group was required to detect a 20% difference in PONV with 80% power and a Type 1 error of 0.05. We included 42 patients in each group to compensate for an expected 10% patient dropout rate.

All statistical analyses were performed using SPSS 23.0 software (SPSS Inc., Chicago, IL, USA). Data were presented as mean ± SD, numbers, and percentages. One way ANOVA was used for comparison of quantitative data. Chi square test and Fisher exact test was applied for comparison of the categorical variables among the groups. A value of *P* < 0.05 was considered statistically significant. Bonferroni method was used to control the type-I error for the multiple comparison among DEX1, DEX2 and Placebo. Thus, 0.05/3 = 0.017 was the significance level for the multiple comparison in this study.

## Results

Of the 126 patients recruited, four patients were excluded due to follow-up loss. We analyzed data from 122 patients (group Placebo, *n* = 41; group DEX1, *n* = 40; group DEX2, n = 41). Patient characteristics were comparable with respect to age, sex, BMI, duration of surgery and anesthesia, LMA removal time and PACU stay time (Table 1). In addition, the numbers of eyes and muscles operated on were similar among the three groups (Table 1).

**Table 1:** Patient demographic and clinical data

	Placebo (*n* = 41)	DEX1 (*n* = 40)	DEX2 (*n* = 41)	*P*
Age (y)	8.24 ± 1.32	8.25 ± 1.06	8.32 ± 1.08	0.951
Sex (Male/female), n	24/17	20/20	19/22	0.526
BMI (kg/ m^2^)	17.07 ± 2.98	16.57 ± 3.91	16.18 ± 2.80	0.272
Duration of surgery (min)	23.43 ± 7.86	22.70 ± 6.61	22.68 ± 6.73	0.938
Duration of anesthesia (min)	31.70 ± 7.58	32.53 ± 8.21	32.48 ± 7.69	0.887
LMA removal time (min)	25.12 ± 8.07	26.28 ± 6.72	27.45 ± 7.02	0.252
PACU stay time (min)	41.25 ± 8.08	41.20 ± 7.25	43.95 ± 8.33	0.104
No. of operated eyes: 1/2	17/24	17/23	10/31	0.160
No. of operated muscles: 1/2/3/4	2/24/6/9	1/33/1/5	1/33/3/4	0.210

Data are expressed as mean ± standard deviation or number

*BMI* body mass index, *LMA* laryngeal mask airway, *PACU* post anesthesia care unit

As shown in Table 2, when multiply comparising among DEX1, DEX2 and Placebo, Bonferroni method was used to control the type-I error and 0.05/3 = 0.017 was the significance level for the multiple comparison in this study. According to the results of Pearson Chi-Square test, the incidence of intraoperative oculocardiac reflex was significantly reduced in the DEX2 group (42%) as compared to the Placebo group (68%) (*p* = 0.0146). However, there was no significant difference between the DEX2 group and the Placebo group based on the results of Yates correction (*p* = 0.026). During the first 24 h post-op, the overall incidence of PONV was significantly lower in the DEX2 group (10%) than in the Placebo group (32%) according to the results of Pearson Chi-Square test (*p* = 0.0142). While there was no significant difference between the two groups based on the results of Yates correction (*p* = 0.029). There was no significant difference among three groups in POV over the entire 24 h after surgery. The incidence of bucking on LMA removal was similar among the three groups.

**Table 2:** Incidence of Oculocardiac reflex, POV, PONV and bucking

	Placebo(*n* = 41)	DEX1(*n* = 40)	DEX2(*n* = 41)	*P*
Oculocardiac reflex	28 (68%)	20 (50%)	17 (42%)^a^	0.045
POV	5 (12%)	3 (8%)	1 (2%)	0.240
PONV	13 (32%)	8 (20%)	4 (10%)^a^	0.048
Bucking	4 (10%)	0 (0)	1 (2%)	0.069

*POV* postoperative vomiting, *PONV* postoperative nausea and vomiting

^a^*P* < 0 .017 vs Placebo group based on Pearson Chi-Square test

**Fig. 1:**
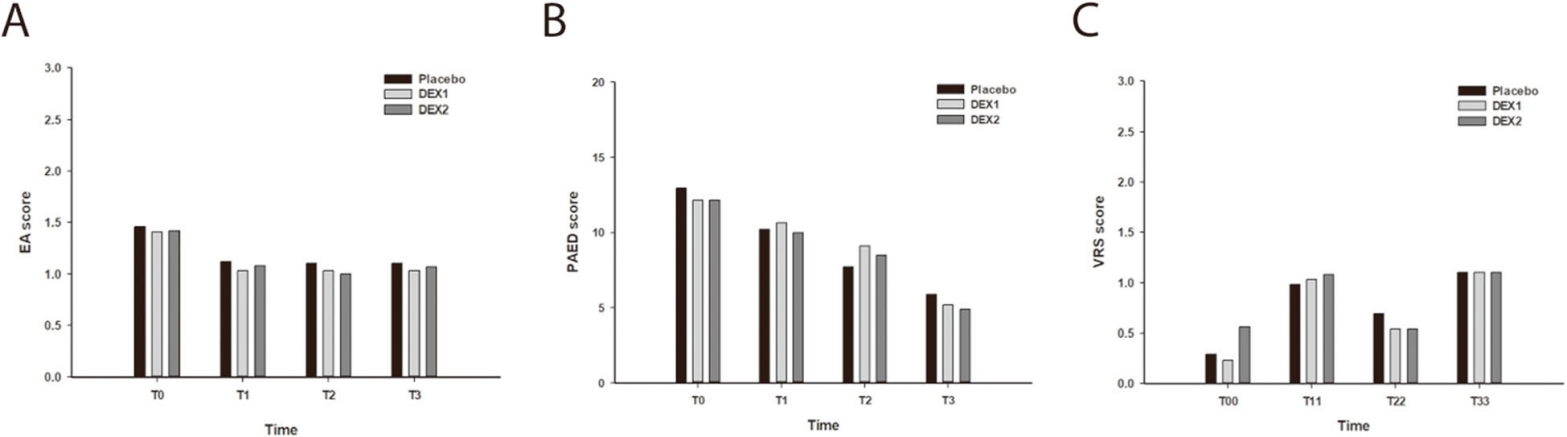
Postoperative agitation and pain profile. EA = emergence agitation; PAED = Pediatric Anesthesia Emergence Delirium; VRS: visual rating scale; T_0_: removing the LMA; T_1_: 5 min after removing the LMA; T_2_: 10 min after removing the LMA; T_3_: 15 min after removing the LMA; T_00_: out of PACU; T_11_: 4 h after operation; T_22_: 12 h after operation; T_33_: 24 h after operation

Figure 1 shows that the PAED scores and EA scores of pediatric patients among three groups were similar at T0, T1, T2, and T3, respectively. There was no significant difference in VRS scores among the three groups during the entire 24 h after surgery.

## Discussion

The present study demonstrated that, for pediatric patients undergoing strabismus surgery under sevoflurane anesthesia, combined use of 0.5 μg/kg DEX reduced the rate of OCR during the operation as well as the incidence of PONV during the 24 h after surgery (based on the results of Pearson Chi-Square test). However, it did not reduce the occurrence of POV during the 24 h after surgery.

As a common surgical operation in children, strabismus surgery carries a high risk of PONV for pediatric patients. There are four risk factors for PONV or POV in pediatric anesthesia: duration of surgery longer than 30 min, age ≥ 3 years, previous PONV or a positive family history, and strabismus surgery [2]. The patients recruited in our study (aged 6–10 years old, undergoing strabismus surgery) are considered highly susceptible for PONV. In attempts to reduce the incidence PONV in patients with risk factors, multiple studies have shown that multimodal antiemetic therapies are more beneficial than a single approach [21, 22].

Previous studies demonstrated that combined use of dexmedetomidine along with balanced anesthesia decreases the incidence of PONV in pediatric patients. Gupta et al. demonstrated that intraoperative use of dexmedetomidine (1 μg/kg loading dose followed by 0.5 μg/kg/h infusion) in children undergoing spinal surgery reduced the incidence of PONV [23]. In addition, Chen et al. reported that administration of dexmedetomidine (1 μg/kg loading dose followed by 0.5 μg/kg/h infusion) decreased the incidence of POV in pediatric patients undergoing strabismus surgery [13]. A meta-analysis examining the effects of premedication with intranasal dexmedetomidine in children demonstrated that patients who received intranasal dexmedetomidine (1 μg/kg intranasal dose) as a premedication experienced a significantly lower incidence of PONV [24]. In the present study, 0.5 μg/kg of intravenously administered dexmedetomidine reduced the occurrence of PONV during the first 24 h after surgery (based on the result of Pearson Chi-Square test). However, 0.3 μg/kg dexmedetomidine was not sufficient to decrease PONV incidence. A meta-analysis focused on dexmedetomidine used for PONV prevention demonstrated that a 0.5–1.0 μg/kg bolus infusion could effectively decrease the incidence of PONV [14]. Compared to these prior studies, the effective dose in our study was relatively low (0.5 μg/kg). This may be due to differing operations, surgery or anesthesia time, ages of recruited patients and combined anesthesia drugs involved in the determination of the dose required to effectively reduce the incidence of PONV.

The beneficial effects of dexmedetomidine include analgesic, sedative and anti-sympathetic actions while avoiding hemodynamic abnormality and respiratory depression [25]. Possible explanations for the success of dexmedetomidine in PONV prevention include multiple mechanisms. The dexmedetomidine-induced opioid-sparing and inhaled anesthetics-sparing effect may contribute to the reduction in PONV [26, 27]. Also, dexmedetomidine decreases noradrenergic activity through reducing sympathetic outflow or inhibiting α_2_ presynaptic in the locus coeruleus which may relate to PONV [28]. In the present study, we found that the incidence of intraoperative OCR was also significantly reduced in the DEX2 group as compared to the Placebo group (based on the results of Pearson Chi-Square test). This suggested an interaction between lower intraoperative OCR incidence and reduced PONV. Previous studies have assessed the relationship between OCR and PONV due to the existence of the oculo-emetic reflex (OER) which is recognized as a trigger of PONV [29, 30].

The optimal dose of dexmedetomidine for achieving anti-emetic effects has not been well documented. According to previous studies, the sedative effect of dexmedetomidine was dose-dependent [31, 32]. In this study, EA and PAED scores were similar among the three groups across all time points during recovery. This may be due to the lower doses of dexmedetomidine we selected (0.3 μg/kg and 0.5 μg/kg loading dose). Additionally, LMA was used as an airway management tool for all three groups and, as previous studies have demonstrated, LMA is more effective than an endotracheal tube in controlling emergence agitation [33, 34]. Thirdly, adequate analgesia (fentanyl and local anesthetics) during the procedure among all three groups reduced the incidence of emergence agitation.

Bradycardia and hypotension are the most common adverse events associated with higher doses of dexmedetomidine [35].The hemodynamic effects of dexmedetomidine are related to the rate of infusion and dose. In our study, the lower doses of dexmedetomidine (0.3 μg/kg and 0.5 μg/kg loading dose) were selected and every drug was given intravenously every 10 min, therefore no significant bradycardia or hypotension were observed during the procedure. Supplemental use of dexmedetomidine (0.5 μg/kg) was administrated during anesthesia induction and assisted in achieving adequate anesthesia depth. Thus, OCR was reduced during the operation.

Our study had several limitations. First, the patients recruited for this study (aged 6–10 years old, undergoing strabismus surgery) are highly susceptible for PONV. Therefore, multimodal antiemetic approach including dexamethasone and droperidol was administered to the three groups in our study. Interactions among the different drugs may be a confounding factor in analyzing the antiemetic effect of dexmedetomidine. Secondly, the selection of muscles that were resected in the operation were not recorded precisely. Previous studies suggested that extraocular muscle involvement was related to the incidence of PONV [36, 37]. We were unable to examine the influence of different muscles on PONV in the present study.

## Conclusions

In conclusion, supplemental use of dexmedetomidine (0.5 μg/kg) in treating pediatric patients undergoing strabismus surgery reduced the rate of OCR and incidence of PONV (based on the results of Pearson Chi-Square test) without lengthening extubation time or recovery time.

## Data Availability

The data sets used and/or analysed during the current study are available from the corresponding author on reasonable request.

## Abbreviations

ASA: American society of anesthesiologists
DEX: Dexmedetomidine
EA: Emergence agitation
OCR: Oculocardiac reflex
PACU: post anesthesia care unit
PAED: Pediatric anesthesia emergence delirium
PCA: patient-controlled analgesia
PONV: Postoperative nausea and vomiting
POV: Postoperative vomiting
VRS: visual rating scale

## References

[1] Höhne C (2014). Postoperative nausea and vomiting in pediatric anesthesia. Curr Opin Anaesthesiol.

[2] Eberhart LH, Geldner G, Kranke P, Morin AM, Schäuffelen A, Treiber H, Wulf H (2004). The development and validation of a risk score to predict the probability of postoperative vomiting in pediatric patients. Anesth Analg.

[3] Joo J, Park S, Park HJ, Shin SY (2016). Ramosetron versus ondansetron for postoperative nausea and vomiting in strabismus surgery patients. BMC Anesthesiol.

[4] Habib AS, Gan TJ (2004). Evidence-based management of postoperative nausea and vomiting: a review. Can J Anaesth.

[5] Madan R, Bhatia A, Chakithandy S, Subramaniam R, Rammohan G, Deshpande S, Singh M, Kaul HL (2005). Prophylactic dexamethasone for postoperative nausea and vomiting in pediatric strabismus surgery: a dose ranging and safety evaluation study. Anesth Analg.

[6] Bicer C, Aksu R, Ulgey A, Madenoglu H, Dogan H, Yildiz K, Boyaci A (2011). Different doses of palonosetron for the prevention of postoperative nausea and vomiting in children undergoing strabismus surgery. Drugs R D.

[7] Sayed JA, MA FR, MO MA (2016). Comparison of dexamethasone or intravenous fluids or combination of both on postoperative nausea, vomiting and pain in pediatric strabismus surgery. J Clin Anesth.

[8] Blaudszun G, Lysakowski C, Elia N, Tramèr MR (2012). Effect of perioperative systemic α2 agonists on postoperative morphine consumption and painintensity: systematic review and meta-analysis of randomized controlled trials. Anesthesiology..

[9] Su F, Hammer GB (2011). Dexmedetomidine: pediatric pharmacology, clinical uses and safety. Expert Opin Drug Saf.

[10] Ibacache ME, Muñoz HR, Brandes V, Morales AL (2004). Single-dose dexmedetomidine reduces agitation after sevoflurane anesthesia in children. Anesth Analg.

[11] Hauber JA, Davis PJ, Bendel LP, Martyn SV, McCarthy DL, Evans MC, Cladis FP, Cunningham S, Lang RS, Campbell NF, Tuchman JB, Young MC (2015). Dexmedetomidine as a rapid bolus for treatment and prophylactic prevention of emergence agitation in anesthetized children. Anesth Analg.

[12] Sun L, Guo R, Sun L (2014). Dexmedetomidine for preventing sevoflurane-related emergence agitation in children: a meta-analysis of randomized controlled trials. Acta Anaesthesiol Scand.

[13] Chen JY, Jia JE, Liu TJ, Qin MJ, Li WX (2013). Comparison of the effects of dexmedetomidine, ketamine, and placebo on emergence agitation after strabismus surgery in children. Can J Anaesth.

[14] Liang X, Zhou M, Feng JJ, Wu L, Fang SP, Ge XY, Sun HJ, Ren PC, Lv X (2015). Efficacy of dexmedetomidine on postoperative nausea and vomiting: a meta-analysis of randomized controlled trials. Int J Clin Exp Med.

[15] Sato M, Shirakami G, Tazuke-Nishimura M, Matsuura S, Tanimoto K, Fukuda K (2010). Effect of single-dose dexmedetomidine on emergence agitation and recovery profiles after sevoflurane anesthesia in pediatric ambulatory surgery. J Anesth.

[16] Ali MA, Abdellatif AA (2013). Prevention of sevoflurane related emergence agitation in children undergoing adenotonsillectomy: a comparison of dexmedetomidine and propofol. Saudi J Anaesth.

[17] Choi EK, Seo Y, Lim DG, Park S (2017). Postoperative nausea and vomiting after thyroidectomy: a comparison between dexmedetomidine and remifentanil as part of balanced anesthesia. Korean J Anesthesiol.

[18] Bedirli N, Akçabay M, Emik U (2017). Tramadol vs dexmedetomidine for emergence agitation control in pediatric patients undergoing adenotonsillectomy with sevoflurane anesthesia: prospective randomized controlled clinical study. BMC Anesthesiol.

[19] Sikich N, Lerman J (2004). Development and psychometric evaluation of the pediatric anesthesia emergence delirium scale. Anesthesiology..

[20] Cole JW, Murray DJ, McAllister JD, Hirshberg GE (2002). Emergence behaviour in children: defining the incidence of excitement and agitation following anaesthesia. Paediatr Anaesth.

[21] Lee SJ, Lee SM, Kim SI, Ok SY, Kim SH, Park SY, Kim MG (2012). The effect of aprepitant for the prevention of postoperative nausea and vomiting in patients undergoing gynecologic surgery with intravenous patient controlled analgesia using fentanyl: aprepitant plus ramosetron vs ramosetron alone. Korean J Anesthesiol..

[22] Riley TJ, McKenzie R, Trantisira BR, Hamilton DL (1998). Droperidol-ondansetron combination versus droperidol alone for postoperative control of emesis after total abdominal hysterectomy. J Clin Anesth.

[23] Gupta N, Rath GP, Prabhakar H, Dash HH (2013). Effect of intraoperative dexmedetomidine on postoperative recovery profile of children undergoing surgery for spinal dysraphism. J Neurosurg Anesthesiol.

[24] Jun JH, Kim KN, Kim JY, Song SM (2017). The effects of intranasal dexmedetomidine premedication in children: a systematic review and meta-analysis. Can J Anaesth.

[25] Iirola T, Ihmsen H, Laitio R, Kentala E, Aantaa R, Kurvinen JP, Scheinin M, Schwilden H, Schüttler J, Olkkola KT (2012). Population pharmacokinetics of dexmedetomidine during long-term sedation in intensive care patients. Br J Anaesth.

[26] Gurbet A, Basagan-Mogol E, Turker G, Ugun F, Kaya FN, Ozcan B (2006). Intraoperative infusion of dexmedetomidine reduces perioperative analgesic requirements. Can J Anaesth.

[27] Lin TF, Yeh YC, Lin FS, Wang YP, Lin CJ, Sun WZ, Fan SZ (2009). Effect of combining dexmedetomidine and morphine for intravenous patient-controlled analgesia. Br J Anaesth.

[28] Whittington RA, Virág L (2006). Dexmedetomidine-induced decreases in accumbal dopamine in the rat are partly mediated via the locus coeruleus. Anesth Analg.

[29] Fry RA (1998). The association between the oculocardiac reflex and post-operative vomiting in children undergoing strabismus surgery. Eye (Lond).

[30] Karanovic N, Carev M, Ujevic A, Kardum G, Dogas Z (2006). Association of oculocardiac reflex and postoperative nausea and vomiting in strabismus surgery in children anesthetized with halothane and nitrous oxide. Paediatr Anaesth.

[31] Phan H, Nahata MC (2008). Clinical uses of dexmedetomidine in pediatric patients. Paediatr Drugs.

[32] Yuen VM (2010). Dexmedetomidine: perioperative applications in children. Paediatr Anaesth.

[33] Lee YC, Kim JM, Ko HB, Lee SR (2011). Use of laryngeal mask airway and its removal in a deeply anaesthetized state reduces emergence agitation after sevoflurane anaesthesia in children. J Int Med Res.

[34] Stevanovic A, Rossaint R, Fritz HG, Froeba G, Heine J, Puehringer FK, Tonner PH, Coburn M (2015). Airway reactions and emergence times in general laryngeal mask airway anaesthesia: a meta-analysis. Eur J Anaesthesiol.

[35] Plambech MZ, Afshari A (2015). Dexmedetomidine in the pediatric population: a review. Minerva Anestesiol.

[36] Simon JW (2010). Complications of strabismus surgery. Curr Opin Ophthalmol.

[37] Lai YH, Hsu HT, Wang HZ, Cheng KI, Wu KY (2014). The oculocardiac reflex during strabismus surgery: its relationship to preoperative clinical eye findings and subsequent postoperative emesis. J AAPOS.

